# Feasibility of a Conditional Knockout System for *Chlamydia* Based on CRISPR Interference

**DOI:** 10.3389/fcimb.2018.00059

**Published:** 2018-02-27

**Authors:** Scot P. Ouellette

**Affiliations:** ^1^Department of Pathology and Microbiology, University of Nebraska Medical Center, Omaha, NE, United States; ^2^Division of Basic Biomedical Sciences, Sanford School of Medicine, The University of South Dakota, Vermillion, SD, United States

**Keywords:** *Chlamydia*, CRISPRi, inducible knockout, reductive evolution, obligate intracellular bacterium

## Abstract

*Chlamydia* is an obligate intracellular bacterium and, as such, has significantly reduced its genome size and content. Although recent advances have allowed for transformation of *C. trachomatis* with an exogenous plasmid, genetic manipulation of *Chlamydia* remains challenging. In particular, the ability to create conditional knockouts has not been developed. This is particularly important given the likelihood that most genes within the small genome of *Chlamydia* may be essential. Here, I describe the feasibility of using CRISPR interference (CRISPRi) based on the catalytically inactive Cas9 variant (dCas9) of *Staphylococcus aureus* to inducibly, and reversibly, repress gene expression in *C. trachomatis*. CRISPRi has been developed and used successfully in a variety of bacterial organisms including *E. coli* and *Mycobacterium tuberculosis*. I first describe the creation of a single plasmid system for CRISPRi in *Chlamydia*, targeted to a non-essential gene, *incA*, that expresses a dispensable inclusion membrane protein. Control transformations of *C. trachomatis* serovar L2 with plasmids encoding only the dCas9, under the control of an inducible promoter, or only the guide RNA (gRNA) targeted to the 5' UTR of *incA*, expressed constitutively, failed to prevent expression of IncA. Importantly, expression of dCas9 alone did not have a deleterious effect on chlamydiae. Transformation of *C. trachomatis* with a plasmid combining the dCas9 and a gRNA targeting *incA* and induction of expression of the dCas9 resulted in the reversible inhibition of IncA expression. Consequently, conditional knockout mediated by CRISPRi is feasible in *Chlamydia*. Potential improvements and experimental concerns in using the system are also discussed.

## Introduction

*Chlamydia* is an obligate intracellular, Gram-negative pathogen with a highly reduced genome. This unique organism differentiates between distinct developmental forms within its host cell while growing within a membrane-bound pathogen-specified parasitic organelle termed an inclusion (Moore and Ouellette, 2014). The inherent challenge of working with an obligate intracellular pathogen has made genetic modification of this bacterium difficult. However, recent advances have made chlamydial genetics not only imaginable but possible. The Clarke lab was the first to describe a method for transforming *Chlamydia trachomatis* with a shuttle plasmid consisting of the endogenous chlamydial plasmid fused to a standard *E. coli* lab vector encoding penicillin resistance (Wang et al., 2011). Penicillin blocks chlamydial cell division and leads to aberrantly enlarged bacteria that are easily distinguished from transformed, penicillin-resistant bacteria (Ouellette et al., 2012). This advance has led to the rapid deployment of a variety of genetic techniques including inducible expression vectors (Wickstrum et al., 2013), translational fusions (Agaisse and Derre, 2013), and directed knockouts (Johnson and Fisher, 2013; Mueller et al., 2016). These tools, along with chemical mutagenesis (e.g., Kari et al., 2011), have significantly advanced our mechanistic understanding of chlamydial microbiology and pathogenesis.

One key tool missing from the chlamydial genetic toolbox is the ability to create conditional knockouts of a target gene via inducible repression or other means. Given the extensive genome reduction through which *Chlamydia* has evolved, it is likely that the majority of chlamydial genes will be essential. Indeed, the genes that have been successfully deleted or rendered otherwise non-functional are mostly associated with virulence or metabolism and thus likely have redundant roles or can be compensated by *in vitro* culture conditions, respectively (e.g., Johnson and Fisher, 2013; Mueller et al., 2016). To study essential genes and their associated systems at a mechanistic level, a means for selectively shutting off their expression is required. Alternatively, a chemical genetic approach could be implemented with the caveat that identifying a target of a small molecule is not a trivial matter (Ouellette and Carabeo, 2010).

The recent explosion of techniques built around the CRISPR platform has resulted in the possibility of using this system to develop a conditional knockout strategy in *Chlamydia*. CRISPR technology relies on the enzymatic cutting activity of the Cas9 in combination with its targeting guide RNA and protospacer adjacent motif (PAM) that specify the site of cleavage. CRISPRi, or CRISPR interference, is predicated on the ability of a catalytically inactive Cas9 variant, dCas9, to recognize its cognate gRNA and bind a target sequence without cleaving it. This results in a steric block to the transcriptional machinery when the target site is located near the 5' end or in the promoter region of the gene of interest (Qi et al., 2013). The original proof-of-concept for CRISPRi was demonstrated in *E. coli* but has since been exported to other systems, including *Mycobacterium tuberculosis* (Choudhary et al., 2015). By transforming an organism of interest with a dCas9-encoding vector under the control of an inducible promoter (with the gRNA typically expressed constitutively), one can selectively block transcription of a target sequence by inducing expression of the dCas9.

Given the success in implementing other genetic tools in *Chlamydia*, I hypothesized that the CRISPRi system would allow for conditional knockouts in *Chlamydia*. A single-plasmid construct with an inducible dCas9 and constitutive gRNA to target a known dispensable gene as a proof-of-concept is presented. This plasmid, and variants encoding only the dCas9 or only the gRNA, were transformed into *C. trachomatis*. Importantly, inducing expression of the dCas9 alone did not impair growth of the transformed strain and nor did it block expression of the target gene. Likewise, the constitutive expression of the guide RNA alone did not block expression of the target gene. However, inducing dCas9 in the presence of the gRNA resulted in the reversible inhibition of the target gene. Consequently, CRISPRi is feasible in *Chlamydia*. Caveats and potential improvements to the system are discussed in the hope that the broader field of *Chlamydia* researchers might develop further applications of this approach.

## Materials and methods

### Plasmid construction

The *dCas9* gene from *Staphylococcus aureus* was PCR-amplified following the manufacturer's guidelines for Phusion DNA polymerase (New England Biolabs, Ipswich, MA) using the plasmid, pX603-AAV-CMV::NLS-dSaCas9(D10A,N580A)-NLS-3xHA-bGHpA (a gift of Dr. F. Zhang; Addgene plasmid #61594), as template and primers 5′- ATATA*ACCGGT***A**TGAAGCGGAACTACATCCTGGGCCT and 5′-ATATT*CGGCCG*TTA**G**CCCTTTTTGATGATCTGAGGGT. The underlined sequences correspond to *Age*I and *Eag*I sites, respectively, and the bolded nucleotide indicates the beginning of the gene specific sequence. The purified PCR product was digested with the indicated enzymes (Fastdigest from Fermentas/ThermoFisher) and ligated into the *Age*I and *Eag*I digested plasmid pL2-LtetO (kind gift of Dr. P.S. Hefty, Univ. Kansas), which was gel-purified and treated with alkaline phosphatase, using T4 DNA ligase (Fermentas). The ligation was transformed into chemically competent *E. coli* XL1 using standard techniques. The resulting colonies were screened for the correct plasmid, pL2-LtetO-Sa_dCas9, which was isolated and sequenced. The gRNA cassette targeting the 5′ region upstream of *incA* was synthesized by Integrated DNA Technologies (Coralville, IA). The gRNA sequence targeting the template strand was 5′-AATTTTTATCATATAAAGCCC (PAM = TAGGAT). A second gRNA sequence targeting the non-template strand, *incA_IGR2*, was also designed and tested: 5′-GGGAGATGGAGGAGTCACGAT (PAM = TAGAGT). For the non-targeting construct, only the gRNA scaffold was expressed. The gRNA transcription was designed to be under the control of a constitutive but weakened promoter driving *dnaK* expression in *Chlamydia* (Schaumburg and Tan, 2003). The sequence of the synthetic gene cassette(s) is listed in Supplementary Material. The gRNA was PCR-amplified using the synthetic gene cassette as template and primers 5′-gtaaattgattgtacaaggTCTTGAACGGTGGAGACG and 5′-aatttcgtctaacttacgTAAAACGAAAGGCCCAGTC. The lowercase letters correspond to the plasmid specific sequences for insertion into *BamH*I-digested pL2-LtetO-Sa_dCas9 via the HiFi DNA Assembly kit (New England Biolabs) according to the manufacturer's instructions and transformed into *E. coli* NEB5α. The resulting colonies were screened for the correct plasmid, pCRISPRi::L2 (incA_IGR, incA_IGR2, or non-targeting), which was isolated and sequenced. For the plasmid pL2-LtetO-Sa_gRNA, the gRNA cassette was PCR-amplified and inserted as above into pL2-LtetO previously digested with *BamH*I.

### Organisms and cell culture

The murine fibroblast cell line McCoy (a kind gift of Dr. H. Caldwell, NIH) was routinely cultured at 37°C with 5% CO_2_ in DMEM (Gibco/ThermoFisher, Grand Island, NY) supplemented with 10% fetal bovine serum (FBS; HyClone, Logan, UT) and 10 μg/mL gentamicin (Gibco/ThermoFisher). The plasmidless (-pL2) strain of *C. trachomatis* serovar L2 was a kind gift of Dr. I. Clarke (Univ. Southampton) and was propagated in McCoy cells as described (Wang et al., 2011) for use in transformations.

### Chlamydial transformation

Transformations were performed as described previously (Mueller and Fields, 2015) using demethylated plasmids prepared from the *dam-/dcm-* strain of *E. coli* from New England Biolabs. Briefly, 2 μg of demethylated plasmid was added to 2.5 × 10^6^ *C. trachomatis* serovar L2 (lacking the endogenous plasmid; -pL2) in Tris-CaCl_2_ buffer (Wang et al., 2011) and incubated at room temperature for 30 min. McCoy cells plated the day before at 10^6^ per well in a six-well plate were subsequently infected with the transformation mix (one well per transformation). One U/mL penicillin (Millipore-Sigma; St. Louis, MO) was added at 8 h post-infection (hpi). Infected cells were harvested at 48 hpi, centrifuged at 20 k × g for 30 min at 4°C, and resuspended in 1 mL of Hank's Balanced Salt Solution (HBSS; Gibco). The suspension was centrifuged for 5 min at 400 × g at 4°C, and the supernatant was added to 1 mL HBSS to infect a new monolayer of McCoy cells. This process of infecting and harvesting infected cells was repeated until wild-type inclusions were visible and the penicillin-resistant bacteria isolated. When possible, organisms were plaque-purified one time as described elsewhere (Matsumoto et al., 1998) with individual plaques being picked from wells that had 5–10 plaques in total.

### Indirect immunofluorescence microscopy

McCoy cells were seeded in 24-well tissue culture plates on coverslips at a density of 70,000 cells per well. The following day, cells were infected with the indicated strains at a multiplicity of infection (MOI) of 0.2–2. At the indicated hours post-infection (hpi), 10–50 nM anhydrotetracycline (aTc) was added or not to induce expression of the dCas9. Infected cells were fixed at the indicated hpi in methanol at room temperature for 5 min. In a subset of samples, the aTc was washed out at 16 hpi, and the cells were subsequently fixed at 24 hpi to monitor recovery of the target protein expression. *Chlamydia* was labeled with a guinea pig primary antibody (kind gift of Dr. E. Rucks, UNMC), and IncA, the target of the CRISPRi, was labeled with a rabbit primary antibody (kind gift of Dr. T. Hackstadt, Rocky Mountain Labs, NIH). Separately, a rabbit antibody against Sa_Cas9 (Abcam, Cambridge, MA) was used to visualize this target in some experiments. Alexa fluor-conjugated goat secondary antibodies (ThermoFisher) were used to detect the primary antibodies. The coverslips were imaged on an epifluorescent upright Olympus BX60 microscope equipped with a Nikon DS-Qi1Mc camera at a magnification of 40x or, alternatively, a Zeiss Imager.Z2 equipped with an Apotome2 and an Axiocam 506 monochromatic camera using a 63x Apochromat objective. Images were processed equally for contrast and brightness in Adobe Photoshop Creative Cloud 2017.

## Results

### Plasmid design for CRISPRi in *Chlamydia*

As originally constructed for *E. coli*, the CRISPRi system relied on the co-transformation of two plasmids: one encoding the dCas9 from *Streptococcus pyogenes* under the control of the Tet promoter and a second encoding the gRNA under the control of a constitutive promoter (Qi et al., 2013). The PAM sequence for *S. pyogenes* Cas9 is NGG. Given the inherent difficulties in transforming *Chlamydia*, the low efficiency of the process, the lack of compatible origins of replication, and the limited antibiotic selection agents, a single-plasmid version of this system was designed. However, although it was possible to repress expression of a target sequence in *E. coli* with the CRISPRi plasmid (data not shown), it was not possible to successfully transform *C. trachomatis* with it. The reason for this is not clear, but one possibility may have been that leaky expression of the *S. pyogenes* dCas9 was not well-tolerated.

**Figure 1:**
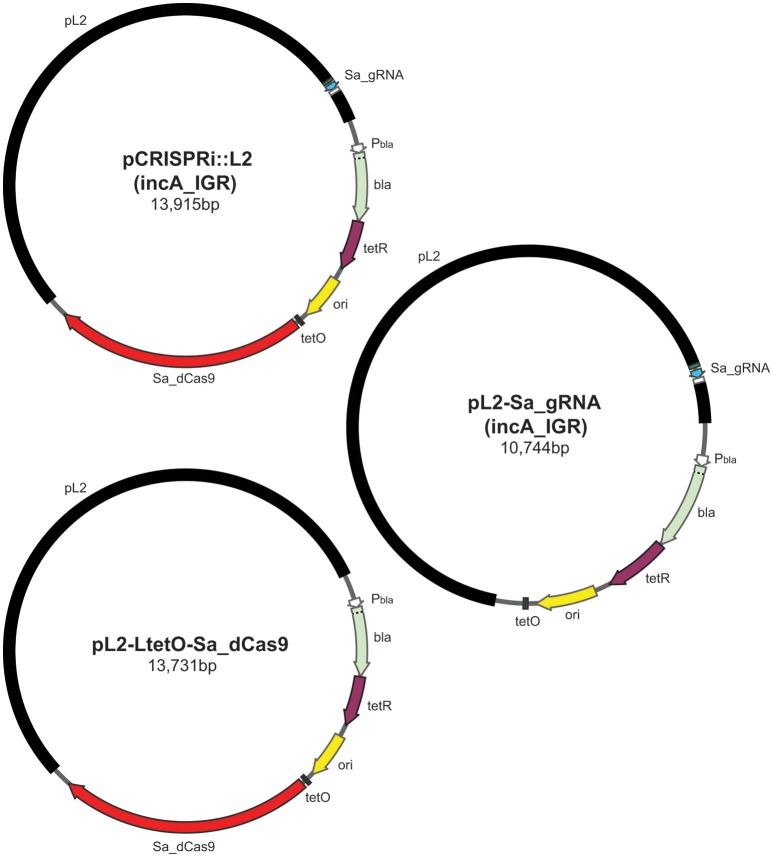
Illustration of plasmids used in the current study and their indicated sizes in basepairs: pCRISPRi::L2 (incA_IGR), pL2-LtetO-Sa_dCas9, and pL2-Sa_gRNA (incA_IGR). The different colors represent different components of the plasmid as indicated: pL2, chlamydial plasmid backbone; Sa_dCas9, *S. aureus* dCas9; tetO, tet operator sites; ori, origin of replication of *E. coli* shuttle vector; tetR, tet repressor; bla, beta-lactamase; Pbla, beta-lactamase promoter; Sa_gRNA, *S. aureus* guide RNA cassette.

Next a single-plasmid CRISPRi construct based on the smaller dCas9 from *Staphylococcus aureus* (Sa_dCas9; Figure 1), which utilizes a different PAM sequence, NNGRRT (Ran et al., 2015), was created. This plasmid encoded the Sa_dCas9 under the control of the Tet promoter and the gRNA under the control of a weakened promoter for *dnaK* that is expressed from an early time post-infection. Because of *Chlamydia*'s developmental cycle, there is no *bona fide* constitutive promoter. As a proof of principle, the Sa_gRNA was designed to target the region upstream from the *incA* start site (incA_IGR). IncA is an inclusion membrane protein that is known to be dispensable and for which there are antibody reagents (Suchland et al., 2000; gift of Dr. T. Hackstadt). IncA was chosen as a target so as to better explore the likelihood of leaky expression that might be deleterious for an essential gene and, indirectly, off-target effects of the system (see below for further discussion on these possibilities). Additional control plasmids were constructed encoding only the inducible Sa_dCas9, only the “constitutive” Sa_gRNA targeting the *incA* intergenic region, or the gRNA scaffold without a targeting sequence and the Sa_dCas9.

### Expression of Sa_dCas9 only, the *incA* gRNA alone, or both Sa_dCas9 and a gRNA scaffold without a targeting sequence does not block IncA expression

**Figure 2:**
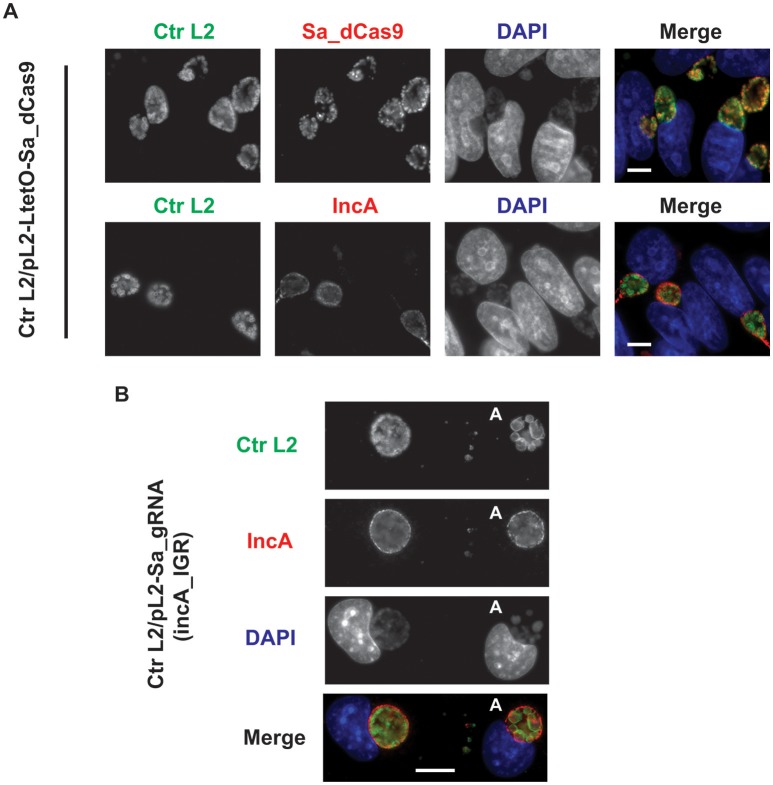
Expression of the *S. aureus* dCas9 or gRNA cassette alone does not block expression of the gRNA target, IncA. **(A)** *C. trachomatis* serovar L2 transformed with the plasmid pL2-LtetO-Sa_dCas9 was used to infect McCoy cells. At 8 hpi, 10 nM anhydrotetracycline (aTc) was added to the cultures, and the infected cells were fixed at 20 hpi. Coverslips were subsequently processed for immunofluorescence analysis as described for the indicated markers. Ctr L2, *C. trachomatis* serovar L2 in green; IncA, inclusion membrane protein A (the target of the gRNA) in red; Sa_dCas9, *S. aureus* dCas9 in red; DAPI, DNA in blue. Note the primary antibodies used to visualize IncA and Sa_dCas9 were both from rabbits so these markers could not be imaged in the same sample. Note the presence of IncA after induction of Sa_dCas9. **(B)** *C. trachomatis* serovar L2 transformed with the plasmid pL2-Sa_gRNA (incA_IGR) was used to infect McCoy cells. Cells were fixed and processed as in **(A)** at 20 hpi in the absence of aTc. The bolded “A” in the images indicates an aberrant inclusion that was not transformed with the plasmid and was, therefore, susceptible to the selection agent, penicillin. Note the presence of IncA in both transformed and untransformed inclusion membranes. The scalebars are equivalent to 10 μm.

To ensure that the Sa_dCas9 had no deleterious effects on chlamydial growth, *C. trachomatis* was transformed with the plasmid encoding only the dCas9. Upon isolation of a pure population of transformants, an experiment was prepared to test the effects of induction of Sa_dCas9 on chlamydiae. McCoy cells were infected with the transformant, and the expression of Sa_dCas9 was induced at 8 h post-infection (hpi), before the first division of the bacterium, by the addition of 10 nM anhydrotetracycline (aTc). Infected cells were fixed at 20 hpi and processed for immunofluorescence for IncA or Sa_dCas9 (both rabbit primary antibodies) in combination with a marker for chlamydiae. As seen in Figure 2A, Sa_dCas9 was clearly present as a cytosolic antigen within chlamydiae, as expected. In addition, IncA staining was clearly visible in replicate samples, thus, as predicted, expression of the dCas9 without the gRNA has no impact on IncA (or presumably any other target sequence). Further, given the large number of organisms present within the inclusions, the Sa_dCas9 was well-tolerated and did not impede chlamydial growth or division. It should be noted that, in the absence of inducer, a faint signal for the dCas9 could be detected by eye in the organisms when visualized on the microscope but was not easily imaged without significantly altering the acquisition settings or artificially enhancing the signal post-acquisition (data not shown, see also Supplementary Figure 1). Therefore, it is likely that the Tet promoter is not completely repressed by the Tet repressor in *Chlamydia* in the absence of inducer.

To ensure that the constitutive expression of the gRNA alone could not act as a small RNA to block *incA* expression, *C. trachomatis* was transformed with the plasmid encoding only the gRNA targeted to the *incA* intergenic region. Although penicillin-resistant transformants were isolated, it was not possible to isolate a pure population as a mixture of penicillin-resistant and—sensitive bacteria were recovered after multiple passages and even after re-infection from a single “resistant” inclusion (i.e., after lysis, adjacent inclusions were of a mixed phenotype). The reason for this apparent plasmid instability is not clear. McCoy cells were infected with the transformant, fixed at 20 hpi, and imaged for IncA. Nevertheless, in bacteria resistant to penicillin, IncA expression was detected (Figure 2B). As expected, IncA was also present on inclusions derived from penicillin-sensitive bacteria. Consequently, constitutively expressed gRNA was insufficient to inhibit expression of the target IncA as a small RNA.

**Figure 3:**
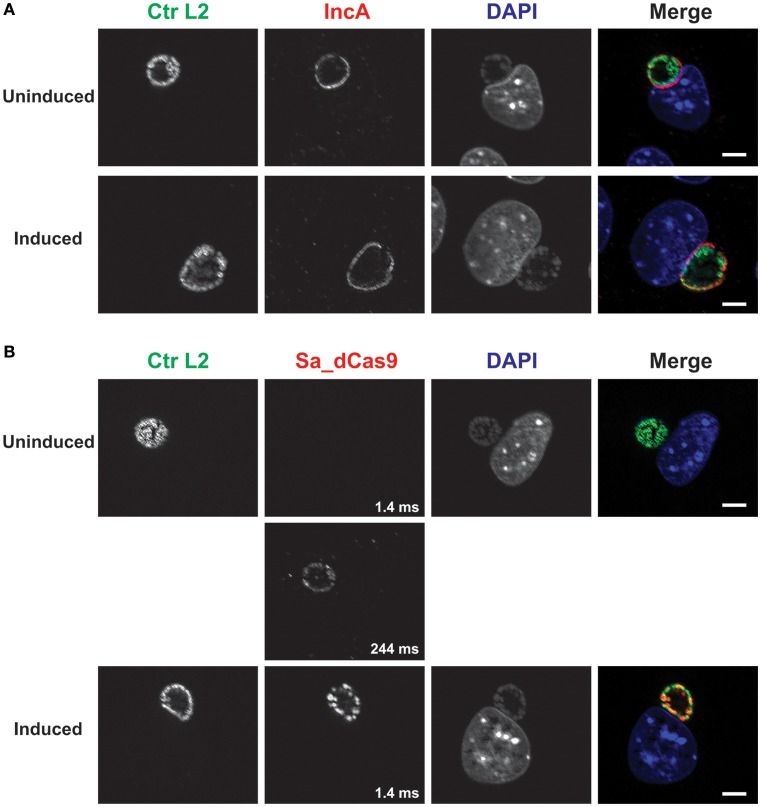
A complete CRISPR interference system without a targeting sequence does not affect IncA expression. **(A)** *C. trachomatis* serovar L2 transformed with the plasmid pCRISPRi::L2-nt (non-targeting) was used to infect McCoy cells. At 12 hpi, 50 nM aTc was added to the cultures, and infected cells were fixed at 20 hpi. Coverslips were subsequently processed for immunofluorescence analysis as described for the indicated markers. Ctr L2, *C. trachomatis* serovar L2 in green; IncA, inclusion membrane protein A in red; DAPI, DNA in blue. Note the presence of IncA in the induced culture. **(B)** Duplicate samples as in **(A)** were processed for immunofluorescence analysis as described for the indicated markers. Ctr L2, *C. trachomatis* serovar L2 in green; Sa_dCas9, *S. aureus* dCas9 in red; DAPI, DNA in blue. Although Sa_dCas9 appears to be absent in the uninduced culture, a long exposure revealed the presence of a low amount of the protein. Exposure times for the different Sa_dCas9 images are noted in the bottom right of the panel. Induction resulted in strong expression of Sa_dCas9 as noted in Figure 2. The scalebars are equivalent to 10 μm.

As another control, a plasmid lacking a targeting gRNA sequence but expressing constitutively the gRNA scaffold and inducible Sa_dCas9 (i.e., non-targeting) was created and used to transform *C. trachomatis* L2. Induction of dCas9 failed to eliminate IncA, as expected (Figure 3). The leakiness of the dCas9 was demonstrated by capturing an extended exposure time. Again, this transformant could not be isolated as a pure population.

### Reversible inhibition of IncA expression by the complete CRISPRi system

**Figure 4:**
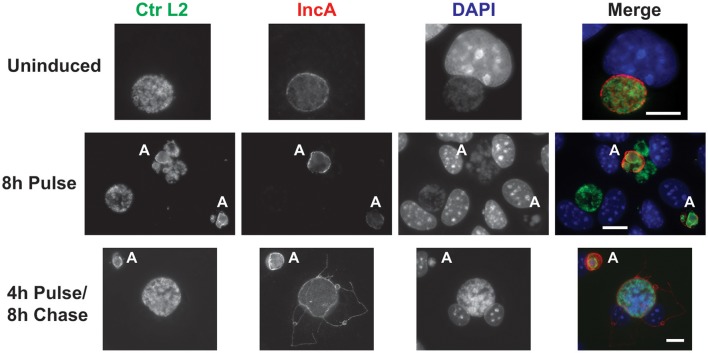
CRISPR interference is sufficient to inducibly, and reversibly, inhibit the target IncA. The panel shows three series of images from McCoy cells infected with *C. trachomatis* serovar L2 transformed with the pCRISPRi::L2 (incA_IGR) plasmid. To determine the efficacy of the system in inducibly repressing a target sequence (IncA), a pulse induction experiment was performed. At 12 hpi, infected cells were treated (8 h Pulse) or not (Uninduced) with 10 nM aTc and fixed at 20 hpi for the top two rows. To determine the reversibility of inhibition of IncA, a pulse-chase was performed. For the bottom row, 10 nM aTc was added at 12 hpi and washed out at 16 hpi. Cells were subsequently fixed at 24 hpi (4 h Pulse/8 h Chase). The markers visualized were as described in the legend of Figure 2. The bolded “A” in the images indicates an aberrant inclusion that was not transformed with the plasmid and was, therefore, susceptible to the selection agent, penicillin. Note the presence of IncA in untransformed but not transformed inclusion membranes after induction of the Sa_dCas9. Likewise, note the presence of IncA after the inducer aTc was washed out of the culture. The scalebars are equivalent to 10 μm.

Given the observations that each component of the CRISPRi system alone was incapable of suppressing expression of the target IncA, the ability of the complete system to inducibly block IncA expression was evaluated. As with the gRNA construct alone, it was not possible to isolate a pure population of penicillin-resistant bacteria. McCoy cells were infected with the transformant and aTc was added at 12 hpi to induce Sa_dCas9. At 16 and 20 hpi, cells were fixed and imaged for the presence of IncA. In a subset of wells, the aTc was washed out at 16 hpi and replaced by fresh medium (with penicillin), and cells were incubated an additional 8 h (24 hpi) before fixation and processing. At both 16 (data not shown) and 20 hpi (Figure 4; 8 h Pulse), and for all penicillin-resistant bacteria, IncA was completely absent, indicating that the CRISPRi system was highly efficient at repressing IncA expression in contrast to samples without aTc where IncA remained on the inclusion (Figure 4). In penicillin-sensitive bacteria (i.e., bacteria without the plasmid conferring resistance), IncA expression was detected, thus showing the dependence of suppression on the CRISPRi plasmid. Similar results were noted using a gRNA targeting a different upstream region of *incA* (incA_IGR2; data not shown). Importantly, removal of the aTc inducer led to the detection of IncA in penicillin-resistant inclusions after 8 h (4 h Pulse/8 h Chase). Therefore, the effects of CRISPRi were reversible. However, the reversibility of IncA repression was not uniform as IncA negative inclusions were visualized in roughly half of penicillin-resistant inclusions after removal of inducer in the pulse-chase samples (data not shown). Presumably, longer chase times would result in a greater percentage of IncA-expressing inclusions albeit with the caveat of leaky dCas9 expression (see below).

## Discussion

Since the Clarke lab's description of a successful method for transformation of *Chlamydia trachomatis* (Wang et al., 2011), there has been a steady development and deployment of genetic tools for this obligate intracellular pathogen. The results described herein suggest one key approach is feasible: conditional, and reversible, knockout by inducible repression using CRISPRi. However, before widespread and quantitative use of this technology can be fully implemented, several barriers must be addressed. It is my hope that the broader field of *Chlamydia* researchers will be able to make further modifications of this technology to address these issues.

Firstly, the issue of plasmid stability must be addressed so that a uniform population of bacteria can be studied. As currently implemented, the CRISPRi system can be used to qualitatively assess the effects of a target protein as long as reagents exist to detect (or not, as the case may be) the target by immunofluorescence. Assessing target gene repression by RT-qPCR or Western blot is not feasible in a mixed population when one is also attempting to determine a phenotypic consequence of repression. Whether the problem is inherent to the plasmid as constructed or represents a different, unrelated issue is not clear. Different chlamydial plasmid backbones (e.g., pL2 and pSW2) were used to construct the CRISPRi plasmid without ameliorating the effects of plasmid instability (data not shown). For the two transformants with plasmid stability issues, the common factor was the Sa_gRNA cassette, suggesting that this might be the problem. However, we have observed other transformants from different constructs without a gRNA to have similar problems (unpublished observation) so it is not clear that the gRNA is itself the cause of the stability issue. Anecdotally, freeze-thawing of the transformant appears to affect the plasmid stability in our hands. The very low efficiency of chlamydial transformation makes these otherwise simple troubleshooting type experiments both time consuming and difficult.

Interestingly, a plasmid encoding wild-type Sa_Cas9 with a gRNA targeted to *incA* intergenic region could not be transformed into *C. trachomatis*. One interpretation of this result is that leaky expression of the Sa_Cas9 in combination with off-target effects due to Sa_gRNA binding elsewhere on the chromosome led to the accumulation of stochastic, deleterious deletion events that precluded the successful isolation of transformants. The 21 bp *incA*-targeting gRNA (incA_IGR) sequence had similarity to four other sequences: *atpI*/CTL0557 (V-type sodium ATPase subunit I; 17 bp match), *glnP*/CTL0384 (ABC transporter permease; 14 bp), *pyrG*/CTL0435 (CTP synthase; 14 bp), and *recB*/CTL0007 (exoribonuclease V beta chain; 15 bp). Multiple sequences matched 11 of the 21 bp gRNA sequence (data not shown). However, none of these sequences had a candidate PAM sequence nearby. Nonetheless, these genes would be the most likely to experience off-target inhibition, which, if true, suggests one or more of these genes is essential in *Chlamydia*. The second gRNA tested, incA_IGR2, displayed no higher than a 13 bp match to *pmpG*/CTL0250 but otherwise displayed the same phenotypes as incA_IGR. Thus, secondly, minimization of off-target effects may need to be addressed. Higher fidelity Cas9 derivatives that accomplish this have been developed for other Cas9 homologs but have not been described for *S. aureus* Cas9 (Chen et al., 2017).

Thirdly, the leaky expression from the Tet promoter must be minimized. Not surprisingly, leakiness of the Sa_dCas9 in combination with the Sa_gRNA was observed to cause inhibition of the target gene expression in a stochastic manner: in the absence of inducer, some inclusions (as much as 50%, depending on the transformant) harboring penicillin-resistant bacteria had little or no detectable IncA. This was observed for multiple transformation attempts of the pCRISPRi::L2 plasmid targeting different regions upstream of *incA*. One solution to address this was attempted by further weakening the promoter driving the Sa_gRNA transcription to further reduce its levels, but this did not eliminate the problem (Supplementary Figure 1). Therefore, another solution is to weaken the Tet promoter elements to eliminate residual Sa_dCas9 expression in the absence of inducer. We are currently designing and testing various promoter constructs to reduce leaky expression.

Taken together, the effectiveness of the CRISPRi system is, therefore, both good and bad. In its current implementation, the system is useful for targeted, microscopy-based studies but is not compatible for population-level analyses. The ability of the Sa_dCas9 to efficiently repress the target sequence suggests that CRISPRi will be extremely useful to study essential gene function if the key barriers described above can be addressed. Other Cas9 homologs are continually being described, and it is possible that a different dCas9 may not have the same limitations as the current design. Finally, if CRISPRi is ultimately not harnessed for *Chlamydia*, then alternative approaches will be required. Given the steady additions to the chlamydial genetic toolbox, I remain confident that our field will continue to advance the methods we use to examine this challenging, and fascinating, organism and that this study will provide a useful platform for further modifications and improvements to CRISPRi in *Chlamydia*.

## Author contributions

SO designed the studies, performed the studies, analyzed the data, and wrote the manuscript.

### Conflict of interest statement

The author declares that the research was conducted in the absence of any commercial or financial relationships that could be construed as a potential conflict of interest.

## References

[B1] Agaisse H. Derre I. (2013). A *C. trachomatis* cloning vector and the generation of *C. trachomatis* strains expressing fluorescent proteins under the control of a C. trachomatis promoter. PLoS ONE 8:e57090. 10.1371/journal.pone.0057090 23441233 PMC3575495

[B2] Chen J. S. Dagdas Y. S. Kleinstiver B. P. Welch M. M. Sousa A. A. Harrington L. B.. (2017). Enhanced proofreading governs CRISPR-Cas9 targeting accuracy. Nature 550, 407–410. 10.1038/nature24268 28931002 PMC5918688

[B3] Choudhary E. Thakur P. Pareek M. Agarwal N. (2015). Gene silencing by CRISPR interference in mycobacteria. Nat. Commun. 6:6267. 10.1038/ncomms7267 25711368

[B4] Johnson C. M. Fisher D. J. (2013). Site-specific, insertional inactivation of *incA* in *Chlamydia trachomatis* using a group II intron. PLoS ONE 8:e83989. 10.1371/journal.pone.0083989 24391860 PMC3877132

[B5] Kari L. Goheen M. M. Randall L. B. Taylor L. D. Carlson J. H. Whitmire W. M.. (2011). Generation of targeted *Chlamydia trachomatis* null mutants. Proc. Natl. Acad. Sci. U.S.A. 108, 7189–7193. 10.1073/pnas.1102229108 21482792 PMC3084044

[B6] Matsumoto A. Izutsu H. Miyashita N. Ohuchi M. (1998). Plaque formation by and plaque cloning of *Chlamydia trachomatis* biovar trachoma. J. Clin. Microbiol. 36, 3013–3019. 9738059 10.1128/jcm.36.10.3013-3019.1998 PMC105103

[B7] Moore E. R. Ouellette S. P. (2014). Reconceptualizing the chlamydial inclusion as a pathogen-specified parasitic organelle: an expanded role for Inc proteins. Front. Cell. Infect. Microbiol. 4:157. 10.3389/fcimb.2014.00157 25401095 PMC4215707

[B8] Mueller K. E. Fields K. A. (2015). Application of beta-lactamase reporter fusions as an indicator of effector protein secretion during infections with the obligate intracellular pathogen *Chlamydia trachomatis*. PLoS ONE 10:e0135295. 10.1371/journal.pone.0135295 26258949 PMC4530969

[B9] Mueller K. E. Wolf K. Fields K. A. (2016). Gene deletion by fluorescence-reported allelic exchange mutagenesis in *Chlamydia trachomatis*. MBio 7:e01817-15. 10.1128/mBio.01817-15 26787828 PMC4725004

[B10] Ouellette S. P. Carabeo R. A. (2010). A functional slow recycling pathway of transferrin is required for growth of *Chlamydia*. Front. Microbiol. 1:112. 10.3389/fmicb.2010.00112 21607082 PMC3095398

[B11] Ouellette S. P. Karimova G. Subtil A. Ladant D. (2012). *Chlamydia* co-opts the rod shape-determining proteins MreB and Pbp2 for cell division. Mol. Microbiol. 85, 164–178. 10.1111/j.1365-2958.2012.08100.x 22624979

[B12] Qi L. S. Larson M. H. Gilbert L. A. Doudna J. A. Weissman J. S. Arkin A. P.. (2013). Repurposing CRISPR as an RNA-guided platform for sequence-specific control of gene expression. Cell 152, 1173–1183. 10.1016/j.cell.2013.02.022 23452860 PMC3664290

[B13] Ran F. A. Cong L. Yan W. X. Scott D. A. Gootenberg J. S. Kriz A. J.. (2015). *In vivo* genome editing using *Staphylococcus aureus* Cas9. Nature 520, 186–191. 10.1038/nature14299 25830891 PMC4393360

[B14] Schaumburg C. S. Tan M. (2003). Mutational analysis of the *Chlamydia trachomatis* dnaK promoter defines the optimal-35 promoter element. Nucl. Acids Res. 31, 551–555. 10.1093/nar/gkg150 12527761 PMC140514

[B15] Suchland R. J. Rockey D. D. Bannantine J. P. Stamm W. E. (2000). Isolates of *Chlamydia trachomatis* that occupy nonfusogenic inclusions lack IncA, a protein localized to the inclusion membrane. Infect. Immun. 68, 360–367. 10.1128/IAI.68.1.360-367.2000 10603409 PMC97142

[B16] Wang Y. Kahane S. Cutcliffe L. T. Skilton R. J. Lambden P. R. Clarke I. N. (2011). Development of a transformation system for *Chlamydia trachomatis*: restoration of glycogen biosynthesis by acquisition of a plasmid shuttle vector. PLoS Pathog. 7:e1002258. 10.1371/journal.ppat.1002258 21966270 PMC3178582

[B17] Wickstrum J. Sammons L. R. Restivo K. N. Hefty P. S. (2013). Conditional gene expression in *Chlamydia trachomatis* using the tet system. PLoS ONE 8:e76743. 10.1371/journal.pone.0076743 24116144 PMC3792055

