# Supplementary material for: Feasibility of a Conditional Knockout System for Chlamydia Based on CRISPR Interference
Source: Front Cell Infect Microbiol. 2018 Feb 27;8:59. doi: 10.3389/fcimb.2018.00059 (PMC5835046; doi:10.3389/fcimb.2018.00059)
Supplement: Supplementary file 1 [file Image1.PDF]

Supplemental Material for Ouellette

Sequence for the Synthetic Gene Cassette for the Sa_gRNA

TC*TTGAAC*GGTGGAGACGGTTTTCT*TATAAT*GACACC**AATTTTTATCATATAAAGCCC**GTTTTA
GTACTCTGGAAACAGAATCTACTAAAACAAGGCAAAATGCCGTGTTTATCTCGTCAACTTGTTG
GCGAGATTTTTCAAATAAAACGAAAGGCTCAGTCGAAAGACTGGGCCTTTCGTTTTA

The italicized sequence corresponds to the weakened P_{dnaK} promoter. The bolded sequence corresponds to the sequence upstream of *incA*. The underlined sequence corresponds to the Sa_gRNA scaffold. The rest of the sequence 3' to the gRNA is the *rrnB1* terminator. The second targeting gRNA, incA_IGR2, had the sequence: 5'-GGGAGATGGAGGAGTCACGAT. For the non-targeting gRNA construct, there was no gRNA upstream of the scaffold.

Supplemental Figure Legend 1. Repression of IncA in the absence of inducer in *C. trachomatis* serovar L2 transformed with pCRISPRi::L2 (incA_IGR). McCoy cells were infected with the transformed strain and fixed at 24hpi to monitor the expression of IncA in the absence of anhydrotetracycline (i.e. uninduced; see also legend to Figure 4 and Discussion). Note that one inclusion is marked with IncA and the other is not. The scalebar is equivalent to 10µm.

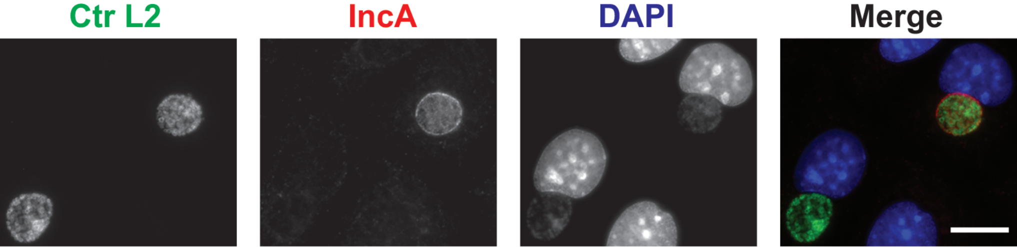

Supplemental Figure 1
